# Evidence for the effect of depth on visual working memory

**DOI:** 10.1038/s41598-017-06719-6

**Published:** 2017-07-25

**Authors:** Jiehui Qian, Jiaofeng Li, Kaiyue Wang, Shengxi Liu, Quan Lei

**Affiliations:** 1 Department of Psychology, Sun Yat-Sen University, Guangzhou, 510000 China; 2 Department of Psychology, University of Minnesota, Minneapolis, 55455 USA

## Abstract

Visual working memory (VWM) is a cognitive memory buffer for temporarily holding, processing, and manipulating visual information. Previous studies have demonstrated mixed results of the effect of depth perception on VWM, with some showing a beneficial effect while others not. In this study, we employed an adapted change detection paradigm to investigate the effects of two depth cues, binocular disparity and relative size. The memory array consisted of a set of pseudo-randomly positioned colored items, and the task was to judge whether the test item was changed compared to the memory item after a retention interval. We found that presenting the items in stereoscopic depth alone hardly affected VWM performance. When combining the two coherent depth cues, a significant larger VWM capacity of the perceptually closer-in-depth items was observed than that of the farther items, but the capacity for the two-depth-planes condition was not significantly different from that for the one-plane condition. Conflicting the two depth cues resulted in cancelling the beneficial effect of presenting items at a closer depth plane. The results indicate that depth perception could affect VWM, and the visual system may have an advantage in maintaining closer-in-depth objects in working memory.

## Introduction

Visual working memory (VWM) is often considered as a cognitive memory buffer for the ‘online’ processing of visual information. It is crucial for visual perception and cognition, e.g., helping to maintain perceptual stability across discontinuation and variations in retinal image as a result of eye, head or body movements. It is also known to be correlated with fluid intelligence^1, 2^. For decades, VWM has been a heated topic and been studied extensively.

One of the putative characteristics of visual working memory is its limited capacity–only three to five items can be stored from a single display^3–8^. However, in reality, we hardly experience any deficiency in temporarily holding and processing visual information. Although we may benefit from selective attention and long-term memory, it is probable that other cognitive factors could affect VWM. For example, studies show that if visual information is properly chunked or grouped, VWM could be enhanced^9–12^. Perceptual grouping, such as Gestalt principles, could make grouped objects appear to ‘belong together’, therefore be processed and stored as a whole. For example, Woodman *et al*. found that proximity and connectedness increase VWM storage^11^; Peterson & Berryhill found that color similarity facilitated VWM but only when the similar stimuli were proximal^10^. Several other grouping principles may benefit VWM performance as well^9, 12–14^. These studies suggest that relevant objects might be grouped together and therefore be remembered better.

Most research on VWM employs visual stimuli presented at a flat screen perpendicular to the line of sight. However, we live in a three-dimensional (3-D) environment. Visual information displayed with 3-D spatial configuration may involve different underlying processing mechanisms from that with a flat, 2-D configuration. Research has investigated the effect of stereoscopic depth on visual processing^15–20^. For example, Nakayama & Silverman found that the visual system could restrict attention in one stereoptic depth plane and ignore distractors at a different depth plane using a visual search task^18^. Viswanathan & Mingolla found that depth cues, such as binocular disparity and occlusion, could aid in tracking moving targets by dividing attention across two 3-D surfaces^20^. These findings suggests that surfaces separated by depth might serve as attentional barriers to restrain objects at the same surface from mixing with objects at a different 3-D surface. Presumably, perceiving an object at a certain depth might automatically allow the visual system to associate a ‘depth tag’ indicating the distance of the object to the observer^21^, therefore objects with the same ‘depth tag’ could be grouped together, and visual processing could be facilitated.

In principle, the same logic could be applied to visual working memory. As perceptual grouping can increase the capacity of VWM, we might also observe beneficial effects on VWM if objects could be ‘grouped’ by depth, i.e., more objects could be retained in VWM across multiple depth planes than within a single depth plane. A few studies had examined this effect, demonstrating mixed results^22, 23^. Xu & Nakayama reported an small increase in number of colored items retained in visual short-term memory (VSTM), when the items were presented on two disparity-defined surfaces compared to that on a single surface^22^. In their study, the authors employed a sequential presentation paradigm rather than simultaneous presentation since their pilot study showed no significant difference between the results of multi-surfaces and that of a single-surface using a conventional change detection paradigm. However, Reeves & Lei found that adding stereoscopic depth hardly affected iconic memory by employing a classic partial report paradigm^23^. The authors suggested a possible strategic difference between the two studies, since observers might successively allocate their attention to the two 3-D surfaces in the former yet no such strategy could be used in the latter.

Because of the apparent differences in the experimental design and the stimuli used in these two studies, whether there is an effect of depth on VWM is still unclear. In addition, although Xu & Nakayama demonstrated an increase in VSTM capacity by introducing disparity-defined depth planes, the authors suggested that it is 3-D surface, not depth information per se, that affected the performance^22^. On the other hand, both studies found no effect of 3-D surface using simultaneous presentation of in-depth stimuli. Xu & Nakayama suggests that it is because that we tend to attend to object in depth serially rather than simultaneously^16^. However, simultaneous presentation of stereoscopic depth could benefit visual processing in many other tasks as discussed above. Is it simply because that simultaneous presentation is not suitable for studying depth effect on VWM, or are there any other confounding factors? It is possible that disparity depth cue alone may not be sufficient to produce depth perception that strong enough to show a significant effect on VWM. Binocular disparity cues are usually more effective at near distances^24–26^, and sometimes could be overridden by contradictory long-range monocular depth cues even at close distance^27, 28^. In the present study, we aimed to test the effect of integrating binocular and monocular depth cues on VWM. By combining the binocular disparity and the monocular relative size cues, we investigated whether making depth perception more salient could clarify the effect of depth on VWM. In addition, by contradicting the two depth cues, we examined whether and how these depth cues could interact with each other to affect the VWM performance.

We employed an adapted change detection paradigm with simultaneous presentation of in-depth stimuli to investigate these questions. As in previous studies, depth perception was created by using stereoscopic disparity. Although in principle disparity could be processed rapidly and the fusion time for stereopsis is about 100 ms with vergence controlled^29^, observers reported feeling difficult to obtain a stable perception of disparity-generated depth with a conventional 50–250 ms presentation of the memory items in a change detection paradigm. Therefore, similar to Reeves & Lei^23^, an frame array indicating the locations of the future memory items was presented prior to the change detection sequence to help the observers acquire the three-dimensional spatial locations of the future memory items. Experiment 1 and 2 tested the effect of a single depth cue, disparity and relative size, respectively. Experiment 3 and 4 examined the joint effect of consistent depth cues and that of conflicting depth cues, respectively.

## Experiment 1: effect of stereoscopic depth on VWM

In this experiment, we investigated the sole effect of stereoscopic depth on VWM. The items in the memory array were randomly divided into two sets and were displayed at two disparity-defined depth planes.

### Method

#### Participants

Fourty-two students from Sun Yat-Sen University with normal or corrected-to-normal vision took part in the experiment for pay. All participants were naive to the purpose of the study. This research was approved by the Sun Yat-Sen University Institutional Review Board (IRB). The study was carried out in accordance with the relevant guidelines and regulations. Written informed consent approved by the IRB was obtained by each participant prior to all the experiments.

#### Apparatus and Stimuli

The stimuli were viewed against a uniform gray background (102.2 *cd*/*m* ^2^) through a Wheatstone stereoscope on a pair of 23-inch HP proDisplay P231 monitors. The display resolution was set to 1600 × 900 pixels, with a refresh rate of 60 Hz. For the typical viewing distance of 70 cm, a pixel subtended approximately 1 arcmin.

**Figure 1:**
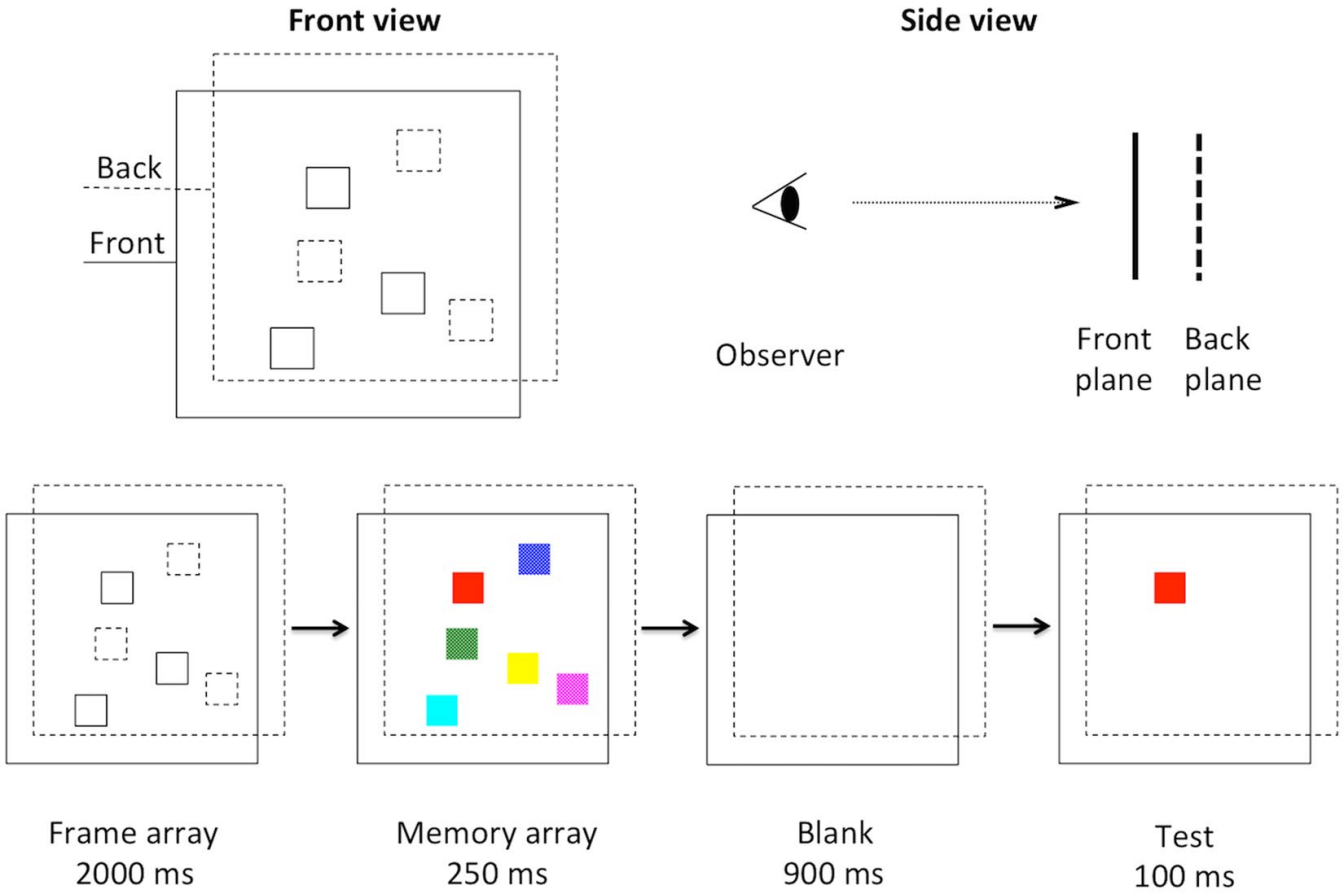
Stimuli and Procedure. Top: the front view and the side view of the frame display. Bottom: task sequences. For demonstration, the frames with solid lines and with dashed lines indicate the front plane and the back plane, respectively. The frame lines were all solid in the formal experiments.

The memory array was composed of a set of colored squares, with their colors randomly selected without replacement from seven highly discriminable colors: red, green, blue, yellow, magenta, cyan, and dark gray. The set size of the colored squares was 4 or 6. The memory items were presented in pseudorandom positions within a 2 × 3 grid subtending 12° × 8°. They were randomly distributed among the six cells, with an angular separation of no less than 1° between any two adjacent items. The two depth planes perpendicular to the line of sight were separated by a relative disparity of 0.1° (Fig. 1). In the crossed-disparity condition, one depth plane was perceived to be in front of the computer monitor screen (the front plane) and the other at the monitor screen (the back plane). In the uncrossed-disparity condition, one was perceived to be behind (the back plane) and the other at the monitor screen (the front plane). Equal number of items were presented at two different planes. Each item subtended 0.65° × 0.65° of visual angle. However, due to the size constancy phenomenon, the items at the back plane appeared to be larger than those at the front. Therefore, we decreased the size of the items at the back plane so that the size of the items at both planes was matched to be the same.

During the test phase, a test item was shown at one of the item locations (test location) in the memory array. Its color would either be the same as the one in the memory array at the test location, or be randomly selected from the colors that had not been previously chosen in the memory array.

#### Procedure

Observers were seated in a dark room to complete the whole experiments. They were trained for a short time (2–5 min) to get acquainted with the stimuli and the task. Each trial began with a fixation cross that subtended 0.3° × 0.3° presented at the center of the screen for 200 ms. In order for the observers to obtain a stable depth perception of the stimuli, we first presented an array of squared frames at the locations of the future memory items for 2000 ms. The memory array that composed of colored squares was then presented for 250 ms. It was followed by a 900 ms blank retention interval and then the test phase. The test item remained on the screen until the observers responded. After the response, an 1000 ms blank intertrial interval was presented before the next trial. A diagram of the task sequences was shown in Fig. 1. The observers were asked to judge whether the color of the test item remained the same as the item at the test location in the memory array. If ‘the same’ was perceived, they pressed the left arrow on the keyboard; otherwise, the right arrow. On 50% of the trials, the color of the test item changed.

In the pilot study, thirty participants were recruited to obtain the change detection accuracy for the ‘one-plane’ control condition, in which all the items were presented at the plane of the monitor screen. This served as a baseline comparison for the Experiment 1–4. In the formal experiment, twenty-one observers participated in the crossed-disparity condition, and another twenty-one participated in the uncrossed-disparity condition. The observers were trained to make sure that they could perceive the disparity-defined depth. Each observer received 120 trials in which the test item were presented at the front depth plane and another 120 trials in which the test item were at the back, yielding a total of 240 trials. The order of the trials were randomized during the experiment.

### Results and Discussion

**Figure 2:**
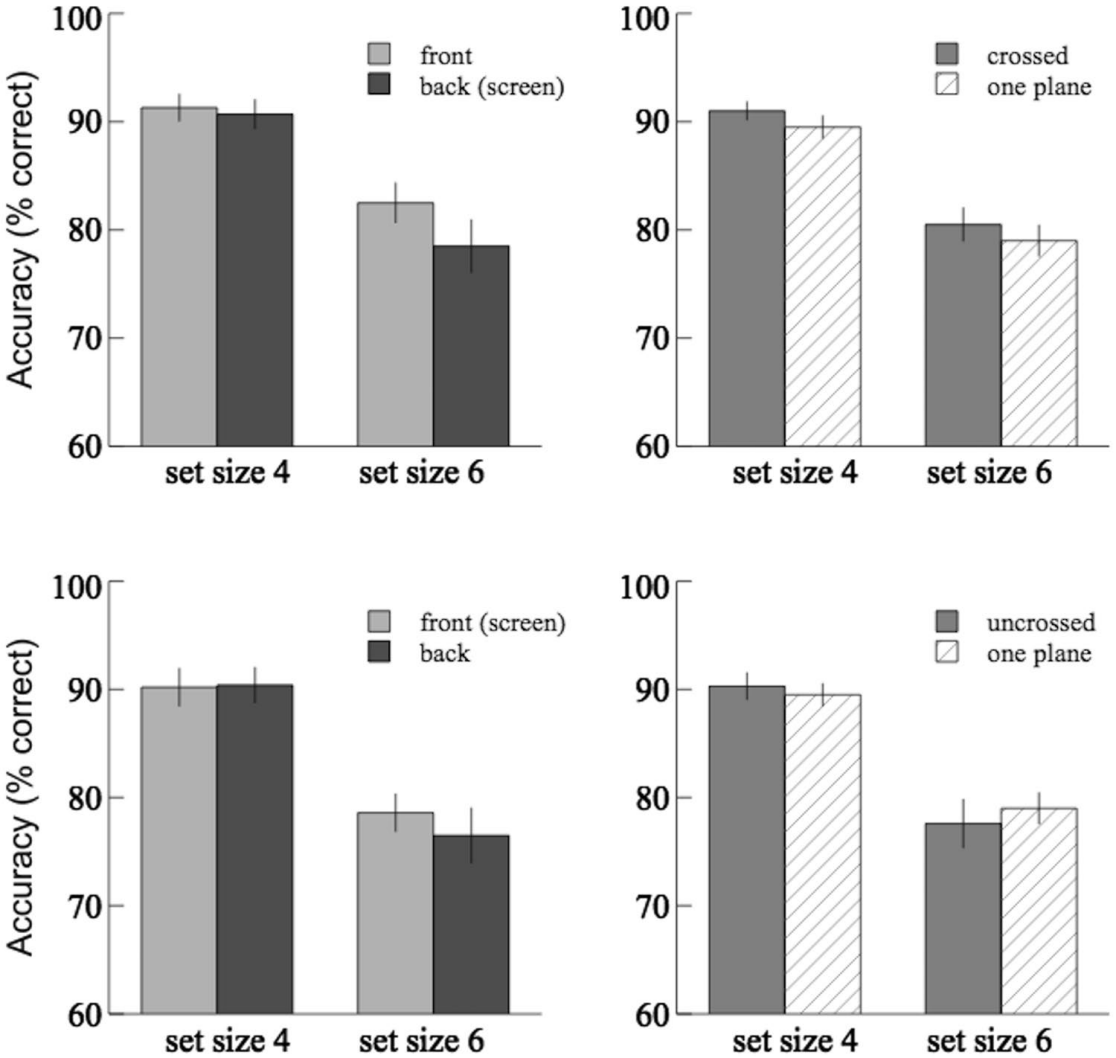
Results of Experiment 1. Top panels: the crossed-disparity condition. Bottom panels: the uncrossed-disparity condition. Comparisons of the average change detection accuracies between the front plane and the back plane were shown on the left; comparisons between the two-plane average and the one-plane were shown on the right.

The pilot study showed that the average change detection accuracy in the ‘one-plane’ control condition was 89.5 ± 1.0% for set size 4 and 79.0 ± 1.4% for set size 6. The results of accuracy in Experiment 1 were shown in Fig. 2; the mean hit and false alarm rates across different set sizes were reported in Table 1. For the crossed- and uncrossed-disparity conditions the results from the front plane were compared with that from the back plane using 2 × 2 (depth order × set size) repeated-measures ANOVA, and the average accuracy of these two depth planes was compared with the results from the one-plane condition using 2 × 2 (depth condition × set size) mixed-design ANOVA. The main effect of set size (4 vs. 6) was significant in both the crossed-disparity ([*F*(1,20) = 29.0, *p* < 0.001, ${\eta }_{p}^{2}$ = 0.59] for repeated measures; [*F*(1,50) = 104.8, *p* < 0.001, ${\eta }_{p}^{2}$ = 0.68] for mixed design) and the uncrossed-disparity ([*F*(1,20) = 116.3, *p* < 0.001, ${\eta }_{p}^{2}$ = 0.85] for repeated measures; [*F*(1,50) = 164.8, *p* = 0.001, ${\eta }_{p}^{2}$ = 0.77] for mixed design) conditions. The main effect of depth order (front vs. back) was not significant in the crossed-disparity condition [*F*(1,20) = 2.96, *p* = 0.10, ${\eta }_{p}^{2}$ = 0.03], nor in the uncrossed-disparity condition [*F*(1,20) = 1.02, *p* = 0.33, ${\eta }_{p}^{2}$ = 0.05]. The main effect of depth condition (two-planes vs. one-plane) was not significant in the crossed-disparity condition [*F*(1,50) = 0.29, *p* = 0.59, ${\eta }_{p}^{2}$ = 0.01], nor in the uncrossed-disparity condition [*F*(1,50) = 0.0, *p* = 0.98, ${\eta }_{p}^{2}$ = 0.0]. No significant interaction effect in any of the above analyses was found. In addition, there was a significant increase in the hit rates of the front plane condition compared to the back plane condition [*t* (20) = 2.39; p < 0.05]. No significant difference in the hit/false alarm rates between the two-planes and one-plane conditions was found.

**Table 1:** Means for hit and false alarm rates at the front and the back planes in Experiment 1–4 and the pilot study.

Experiment	Hit rates		False alarms	
	Front	Back	Front	Back
pilot	0.80 (0.03)	—	0.11 (0.03)	—
1	0.79 (0.03)	0.73 (0.04)	0.08 (0.02)	0.09 (0.02)
2	0.73 (0.04)^+^	0.74 (0.04)^−^	0.07 (0.02)^+^	0.06 (0.02)^−^
3	0.79 (0.03)	0.79 (0.03)	0.06 (0.02)	0.11 (0.02)
4	0.75 (0.04)	0.75 (0.04)	0.08 (0.02)	0.06 (0.01)

Note: ^+^large size condition; ^−^small size condition.

Presenting the memory items at two disparity-defined depth planes does not result in significant change in accuracy compared to presenting them at one plane. This is consistent with the previous findings^22, 23^ that adding stereoscopic depth does not seem to contribute to visual short-term memory if the different depth planes are displayed simultaneously. However, Fig. 2 shows that the performance at the front plane tends to be better than that at the back for set size 6, especially in the crossed-disparity condition, where the front plane is closer to the observer than the plane of fixation (back plane). Additionally, compared to the back plane condition, a significant increase in the hit rates of the front plane condition was found. We calculated an estimate of the VWM capacity, Cowan’s K (*K* = set size × (hit rate - false alarm rate); Cowan, 2001, adapted from Pashler, 1988), and found that the average capacity for set size 6 was 3.9 at the front plane compared to 3.4 at the back plane in the crossed-disparity condition, and was 3.4 at the front compared to 3.1 at the back in the uncrossed-disparity condition. The effect size, *partial η* ^2^, is 0.13 in the crossed-disparity condition and 0.05 in the uncrossed-disparity condition. Given the small effect size, we suspected that there might exist a weak difference in the VWM performance and the estimated capacity between the front plane and the back plane, and the lack of significance might be due to an insufficient sample size. Furthermore, this weak effect might be averaged out across depth, resulting in no significant difference in performance between the two-planes condition and the one-plane condition.

## Experiment 2: effect of relative size on VWM

In Experiment 1, we found no significant effect of depth using binocular disparity cues. However, other depth cues, such as monocular depth cues, could also affect depth perception and therefore might influence VWM. Experiment 2 investigated the effect of relative size on visual working memory.

### Method

#### Participants

Twenty students from Sun Yat-Sen University with normal or corrected-to-normal vision took part in the experiment for pay. All participants were naive to the purpose of the study.

#### Stimuli and Procedure

The experimental procedure was identical to Experiment 1, except the following changes. There was no disparity applied and the stimuli were presented at the monitor screen. The size of the memory items either subtended 0.65° × 0.65° of visual angle (small) or 1.30° × 1.30° (large), and remained the same throughout the trial. There was equal number of items being small and large. As in Experiment 1, the observers were asked to indicate whether the color of an item had changed at the test location. Each observer received 120 trials in which the test item was small and another 120 trials in which the test item was large, yielding a total of 240 trials. The order of the trials were randomized during the experiment.

### Results and Discussion

**Figure 3:**
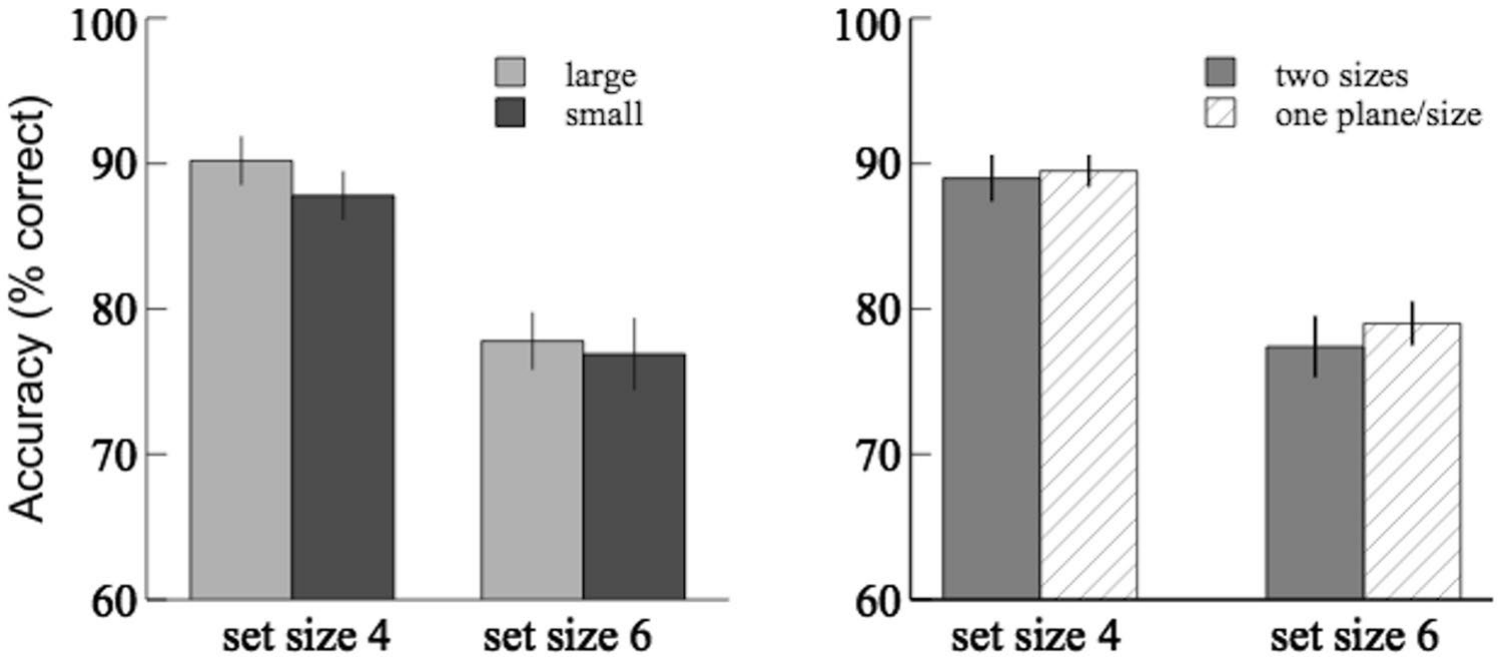
Results of Experiment 2. Comparisons of the average change detection accuracies between the large items and the small items were shown on the left; comparisons between the two-sizes condition and the one-plane/size condition were shown on the right.

During the practice trials, the observers reported that no apparent depth was perceived for the stimuli. It is understandable since the relative size cue alone in static images may be insufficient to induce depth perception. However, this manipulation may still facilitate VWM since items with the same size tend to group together. Therefore, here we investigated whether VWM could be affected by size similarity grouping. The results of accuracy were shown in Fig. 3; the mean hit and false alarm rates were reported in Table 1. The accuracy of the large test items were compared with that of the small items using 2 × 2 (item size × set size) repeated-measures ANOVA, and the average accuracy of the two sizes was compared with that of the ‘one-plane’ control condition in the pilot study using 2 × 2 (grouping condition × set size) mixed-design ANOVA. The main effect of set size was significant ([*F*(1,19) = 41.4, *p* < 0.001, ${\eta }_{p}^{2}$ = 0.69] for repeated measures; [*F*(1,49) = 136.0, *p* < 0.001, ${\eta }_{p}^{2}$ = 0.74] for mixed design). There was no significant main effect of item size [*F*(1,19) = 1.98, *p* = 0.18, ${\eta }_{p}^{2}$ = 0.09], or grouping condition [*F*(1,49) = 0.43, *p* = 0.52, ${\eta }_{p}^{2}$ = 0.01]. No significant interaction effect was found in any of the above analyses. In addition, there was no significant difference in the hit/false alarm rates between the two-sizes condition and one-plane/size conditions, nor between the large- and small-item conditions. The results show that the difference in relative size of the memory items does not result in significant difference in the change detection accuracy, suggesting no effect of size similarity grouping on VWM in this experiment.

## Experiment 3: effect of congruent depth cues on VWM

The results of Experiment 1 and 2 show that neither disparity or relative size alone could affect the change detection performance. However, it is possible that the depth perception in the previous experiments was too weak to result in a significant effect. In Experiment 3, we combined the disparity and the relative size depth cues to make depth perception more salient, and investigated the effect of depth-cue integration on VWM.

### Method

#### Participants

Nineteen students from Sun Yat-Sen University with normal or corrected-to-normal vision took part in the experiment for pay. All of them were naive to the purpose of the study.

#### Stimuli and Procedure

**Figure 4:**
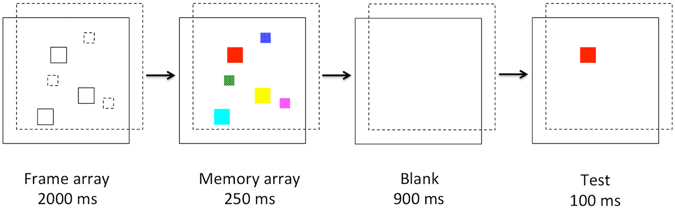
The task sequences in Experiment 3. Half of the items were of a large size at the front depth plane, and half of a small size at the back plane. The frames with solid lines indicates the front plane, the frames with dashed lines indicates the back plane. The frame lines were all solid in the formal experiments.

The experimental procedure was identical to the crossed-disparity condition in Experiment 1, except the following changes. In the front plane, the size of the memory items subtended 1.30° × 1.30° (large); in the back plane, the size of the items subtended 0.65° × 0.65° (small). Since the disparity cues and the relative size cues indicated the same direction of depth, we termed it the ‘congruent’ condition. Their size remained unchanged throughout the trial. There was equal number of items being large while positioned at the front, and being small while positioned at the back (see Fig. 4). The task remained the same as in Experiment 1. Each observer received a total of 240 trials, with 120 trials in which the test item was large and presented at the front. The order of the trials were randomized during the experiment.

### Results and Discussion

**Figure 5:**
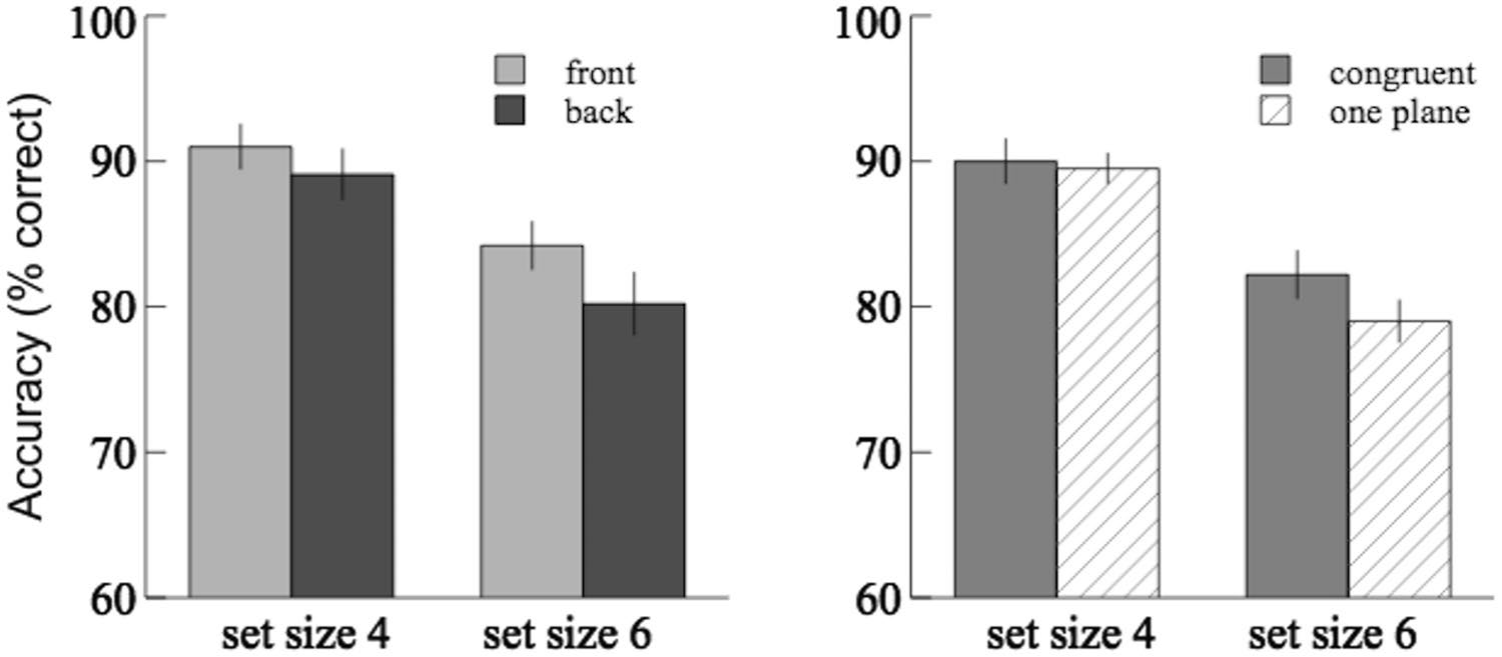
Results of Experiment 3. Comparisons of the average change detection accuracies between the front plane and the back plane were shown on the left; comparisons between the ‘congruent’ two-planes condition and the control condition were shown on the right.

The results of accuracy were shown in Fig. 5; the hit and false alarm rates were reported in Table 1. The accuracy of the front plane was compared with that of the back plane using 2 × 2 (depth order × set size) repeated-measures ANOVA, and the average accuracy of these two planes was compared with that of the ‘one-plane’ control condition in the pilot study using 2 × 2 (depth condition × set size) mixed-design ANOVA. The main effect of set size was significant ([*F*(1,18) = 61.85, *p* < 0.001, ${\eta }_{p}^{2}$ = 0.78] for repeated measures; [*F*(1,48) = 115.25, *p* < 0.001, ${\eta }_{p}^{2}$ = 0.76] for mixed design). The main effect of depth order was significant [*F*(1,18) = 5.31, *p* < 0.05, ${\eta }_{p}^{2}$ = 0.23]. The main effect of depth condition was not significant [*F*(1,48) = 0.88, *p* = 0.35, ${\eta }_{p}^{2}$ = 0.02]. No significant interaction effect was found in any of the above analyses. In addition, there was no significant difference in the hit/false alarm rates between the two-planes and one-plane conditions, but there was a significant decrease in the false alarm rates of the front plane condition compared to the back plane condition [*t*(18) = 3.57; *p* < 0.01].

The results of Experiment 3 showed that the change detection accuracy for the front items was significantly higher than that for the back items, suggesting that the capacity of VWM may differ for objects at different depth planes. It is possible that objects closer to an observer are prioritized for encoding and their features are retained better in VWM. The results confirmed our previous speculation that the lack of a difference between the front and the back planes in Experiment 1 may have resulted from an inadequate depth perception. In Experiment 3, combining disparity and relative size cues created a more vivid and reliable depth perception. In addition, there was a significant decrease in the false alarm rates of the front plane condition compared to the back plane condition, but no significant difference in the hit rates was found. This suggests a higher sensitivity in detecting changes at the front plane. However, the poorer performance at the back plane may cancel out the beneficial effect observed for the front items, therefore no significant difference was found between the two-planes condition and the one-plane condition.

## Experiment 4: effect of incongruent disparity and relative size cues

Experiment 3 shows that the depth perception created by integrating congruent depth cues could enhance the change detection performance for the items closer to the observers. In order to further test the effect of these two depth cues on VWM performance, in Experiment 4, we employed the effect of incongruent depth cues. While the disparity cue indicated one direction of depth, the relative size cue indicated the opposite. The two depth cues in conflict resulted in a less reliable depth perception. By this manipulation, we could compare the change detection performance between the congruent and the incongruent depth-cues conditions, therefore to clarify how disparity cue and relative size cue are integrated to produce a depth effect (or the lack thereof) on VWM.

### Method

#### Participants

Sixteen students from Sun Yat-Sen University with normal or corrected-to-normal vision took part in the experiment for pay. All of them were naive to the purpose of the study.

#### Stimuli and Procedure

The experimental procedure were identical to Experiment 3, except that: in the front plane, the size of the memory items subtended 0.65° × 0.65° (small); in the back plane, the size of the items subtended 1.30° × 1.30° (large). Since the disparity and the relative size cues indicated different direction of depth, we termed it the ‘incongruent’ condition.

### Results and Discussion

**Figure 6:**
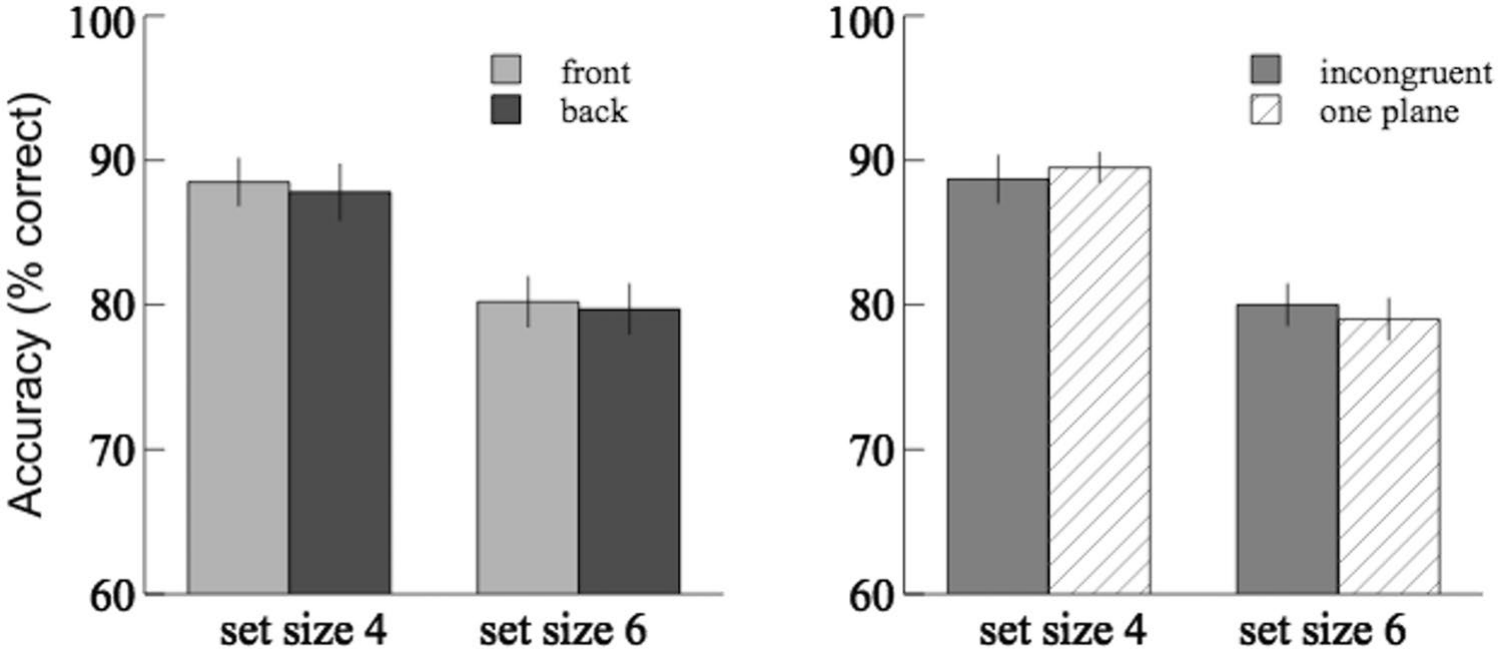
Results of Experiment 4. Comparisons of the average change detection accuracies between the front plane and the back plane were shown on the left; comparisons between the ‘incongruent’ two-planes condition and the control condition were shown on the right.

The results of accuracy were shown in Fig. 6; the hit and false alarm rates were reported in Table 1. The accuracy of the front plane was compared with that of the back plane using 2 × 2 (depth order × set size) repeated-measures ANOVA, and the average accuracy of these two planes was compared with that of the ‘one-plane’ control condition in the pilot study using 2 × 2 (depth condition × set size) mixed-design ANOVA. The main effect of set size was significant ([*F*(1,15) = 21.83, *p* < 0.001, ${\eta }_{p}^{2}$ = 0.59] for repeated measures; [*F*(1,45) = 101.41, *p* < 0.001, ${\eta }_{p}^{2}$ = 0.69] for mixed design). The main effect of depth order was not significant [*F*(1,15) = 0.38, *p* < 0.55, ${\eta }_{p}^{2}$ = 0.03]. The main effect of depth condition was not significant [*F*(1,45) = 0.03, *p* = 0.87, ${\eta }_{p}^{2}$ = 0.001]. No significant interaction effect was found in any of the above analyses. The results suggest that the saliency of depth perception is critical for the depth effect on VWM to occur. Conflicting the disparity and the relative size cues caused the depth perception to be less reliable, therefore the beneficial depth effect for the front items was not observed in this case. Comparing the results from Experiment 1, 3, and 4, the effect size of depth order (front vs. back), ${\eta }_{p}^{2}$, was 0.15 (the crossed-disparity condition), 0.23 and 0.03, respectively. This indicates that the depth effect is greater as the depth perception becomes more salient and realistic. Furthermore, the effect size of depth in this experiment is much smaller than that of Experiment 1, suggesting that the cue combination involves a summation rather than a winner-take-all mechanism.

## General discussion

The present study investigated the effect of depth perception on visual working memory by employing an adapted change detection paradigm. The depth perception was created by disparity cue or a combination of disparity and relative size cues. The four experiments showed that presenting the items in stereoscopic depth (Experiment 1) or contrasting items by relative size (Experiment 2) hardly affected VWM capacity. However, combining congruent depth cues resulted in significantly larger VWM capacity for perceptually closer items than that for farther items (Experiment 3). This effect disappeared as the two depth cues were in conflict, suggesting that combining the incongruent depth cues may balance out the effect of each depth cue (Experiment 4).

Ecologically, a more natural and realistic scene may facilitate visual processing, since our visual system is optimized for processing stimuli that are familiar to us. Evidence showed that familiarity could improve VWM performance and the estimated capacity^30^. Therefore, in principle, displaying visual information in a 3-D space simultaneously may as well facilitate VWM. However, Experiment 1, 3 and 4 showed that the change detection accuracy does not significantly differ between the two-planes condition and the one-plane condition. This is consistent with the findings of Xu & Nakayama and Reeves & Lei using simultaneous presentation of depth^22, 23^. Interestingly, in Experiment 3, the performance improved for the items presented at the front plane compared to those at the back. We also observed the same tendency at set size 6 in Experiment 1, although the repeated-measures ANOVA showed no significant difference. It is possible that the visual system allocates more cognitive resources to the front items than the back items during the encoding phase, and therefore the front items are remembered better. We speculate that the lack of main effect of depth (front vs. back) in Experiment 1 might be due to several possibilities. First, the advantage of the front stimuli might be in effect regardless of the memory load, but only to be obscured by the results at set size 4, where the task might be too easy to detect any accuracy difference. But since the accuracy at set size 4 in Experiment 1 does not reach a performance ceiling, this is an unlikely explanation. Second, the ‘front advantage’ may only occur when the memory load is high and the task is difficult enough to allow the stimuli competing for resources. In other words, the front and the back items may receive equal resources at set size 4; however, when the load of task exceeds the available resources at set size 6, the items at the front plane would get more resources and therefore achieve better performance. Because the effect is relatively small, with the current sample size it is insufficient to obtain a significant difference. The results also show that the effect size in the crossed-disparity condition, where the front plane is closer to the observer than the plane of fixation (back plane), is larger than that in the uncrossed-disparity condition. This suggests that absolute depth perception may indeed matter. As an indicator of VWM capacity, Cowan’s K showed that the average capacity for set size 6 was 3.9 at the front plane compared to 3.4 at the back (screen) plane in the crossed-disparity condition, and was 3.4 at the front (screen) compared to 3.1 at the back in the uncrossed-disparity condition. Therefore, we suggest that depth perception does contribute to VWM, but that the advantage for the front items is canceled by the disadvantage for the back items when averaging the performance at the front and the back. This could as well be a plausible explanation for the lack of an effect between the multi-planes and the one-plane performance reported in Xu & Nakayama’s pilot study^22^ and Reeves & Lei^23^.

Another possibility for the improved performance at the front plane is enhanced attention for perception rather than more resources for better encoding in VWM. Because spreading attention over 3-D space simultaneously could be difficult, one may use a strategy to explore the environment. Ecologically, objects presented closer to us may provide more important and relevant information, therefore it is reasonable to allocate more attention to the front plane at first and then switch attention to the back. In this case, the better performance we observed at the front plane may reflect an improved attentional perception. However, mixed results were reported regarding the attention shift in 3-D space. Some studies showed that switching attention among locations within the same plane is easier than between different 3-D surfaces^16, 31^, and far-to-near attention shifts are faster than near-to-far shifts^32, 33^. But several studies found no difference between the time course of within- and between-plane attention shifts using a spatial cuing task, and therefore implying no cost for switching attention in depth^34, 35^. In addition, He & Nakayama showed that it is 3-D surface rather than depth per se that influences the distribution of visual attention^16^. Because of these inconsistencies, it is difficult to be conclusive about whether and how allocation of attention contributes in our study.

Our study used binocular disparity to create depth perception in Experiment 1, 3 & 4, and used relative size to provide consistent and inconsistent depth cue in Experiment 3 & 4, respectively. We found that disparity cue alone produces little or no effect on VWM. Relative size, being a feature of the memory items by itself, can also serve as a depth cue. As a feature, size could be subject to Gestalt grouping principle–similarity grouping, i.e., the items with the same size tend to group together. However, Experiment 2 found no effect of size grouping. It is possible that when size grouping cooperates with the disparity depth cue, it may affect VWM by facilitating a separation between the items at the front plane and the back plane, although this seems to be unlikely given the lack of an depth order effect in Experiment 4 where size grouping was also present. Nevertheless, we cannot rule out the possibility that large items at the front depth plane receive more resources or attention compared to small items at the back, therefore VWM capacity is higher for these items. On the other hand, when relative size serves as a depth cue and combines with consistent disparity cue, it can greatly enhance depth perception. As a result, a vivid depth perception could facilitate VWM at the front plane that is closer to the observer, while conflicting these depth cues resulted in an unstable depth perception and the effect diminished. This suggests a summation effect of conflicting cues rather than a winner-take-all effect on VWM. However, it is still under debate whether the visual system adopts a simple weighted averaging of the cues or cue dissociation strategy. Regan & Beverley proposed that the monocular and binocular cues combined according to a weighted-sum model^36^. They found that a motion-in-depth perception produced by changing size could be canceled by an opposed change in relative disparity. Heuer confirmed that when the two cues have the same sign they combine by simple summation^37^. However, when one cue signalled an approaching object and the other a receding object the cues rivalled rather than combined with either one dominating. Howard *et al*.’s study also indicates that the conflicting cues rivalled rather than combined^38^. In a series of our previous studies^39–41^, results showed that optic flow alone provided a dominating percept of distance change that prevailed over many of the conflicting depth cues. Landy & Brenner concluded in a review that cue combination of stereo and motion could improve 3-D shape estimates under certain restricted circumstances, but rarely contributes to distance estimates^42^. The divergence in these findings indicates that the mechanism of cue interaction may be different depending on the task and experimental settings.

## Conclusion

Our study found no significant difference in the VWM performance between the two-planes condition and single-plane condition, however, a significant better performance on the perceptually closer-in-depth items was observed than that of the farther items when combining the coherent binocular and monocular depth cues. This effect could not be due to a grouping effect of depth, but rather suggests a difference in allocating attention or cognitive resources between different depth planes.
